# Implementation and outcomes of a novel occupational therapy service in a nursing home

**DOI:** 10.1111/1440-1630.70089

**Published:** 2026-04-27

**Authors:** Brie Bobinskas, Alice Pashley, Helen Holloway, Claire Pearce, Diane Gibson, Nathan D'Cunha, Kasia Bail, Stephen Isbel

**Affiliations:** ^1^ School of Rehabilitation and Exercise Sciences, Faculty of Health University of Canberra Bruce Australian Capital Territory Australia; ^2^ Centre for Ageing Research and Translation, Faculty of Health University of Canberra Bruce Australian Capital Territory Australia; ^3^ Faculty of Health University of Canberra Bruce Australian Capital Territory Australia; ^4^ School of Nursing University of Canberra Bruce Australian Capital Territory Australia

**Keywords:** allied health, dementia, engagement, multidisciplinary teams, nursing home, occupational therapy, residential aged care, transdisciplinary care

## Abstract

**Introduction:**

Occupational therapy plays a vital role in enhancing engagement and quality of life for people living in nursing homes. However, in Australia, funding for occupational therapy in nursing homes is limited, and its scope is restricted. The Enhancing Allied Health for Older People (EAHOP) trial aimed to assess the feasibility and impact of embedding a multidisciplinary allied health model, including occupational therapy, in a nursing home. This paper describes the occupational therapy implementation and outcomes of the EAHOP trial.

**Methods:**

A convergent mixed‐evaluation was conducted. Twenty‐seven residents at an Australian nursing home received occupational therapy by occupational therapists and occupational therapy students. The Canadian Occupational Performance Measure (COPM) was used to set goals and evaluate changes in occupational performance. Data included median therapy minutes per resident and service occasions. Qualitative data were gathered using semi‐structured and unstructured interviews and analysed using content analysis.

**Consumer Involvement:**

The EAHOP trial included a Stakeholder Committee that included residents of the nursing home who were participants in the trial. This group advised the research team on all aspects of the intervention throughout the trial.

**Results:**

Residents received a median of 895 minutes of occupational therapy and 203 minutes from allied health assistants for a 1‐to‐36‐week programme where the intervention period varied according to individual need. This equated to a median of 3.9 minutes/resident/day. COPM scores showed clinically meaningful improvements in performance and satisfaction. Interventions addressed diverse occupational performance issues across self‐care, productivity, and leisure. Qualitative findings supported these outcomes, highlighting the benefits of the service and identifying barriers and implementation challenges.

**Conclusion:**

Embedding a comprehensive occupational therapy service within a multidisciplinary team model is both feasible and beneficial in a nursing home. The improvements in occupational performance and satisfaction, along with the diversity of interventions required, highlight the important contribution of occupational therapy in a nursing home.

## PLAIN LANGUAGE SUMMARY

Occupational therapy may help older people in nursing homes manage daily tasks more easily and safely. In Australia, occupational therapy currently has only a small role in these homes. Researchers from the University of Canberra ran a study called *Enhancing Allied Health for Older People* to see if a team of health workers in a nursing home would help. The study was held in a nursing home in Australia, with 27 residents receiving occupational therapy to do activities that were important to them. Each person's progress was measured by listing key activities and rating how well they could do them before and after support.

Each resident got 895 minutes of occupational therapy and 203 minutes of help from allied health assistants based on their need and wishes. Researchers also talked to family members, staff and students to understand if occupational therapy support was useful and to know of any challenges. The study showed that occupational therapy in a nursing home can make it easier for people to engage in occupations and improve their quality of life.

**Key Points for Occupational Therapy**

- Occupational therapy is important for people living in nursing homes.
- In Australia, occupational therapists are not working to their potential scope of practice in nursing homes.
- A diverse range of occupational therapy can be delivered in nursing homes to improve occupational performance.

## 1 INTRODUCTION

Australia's population is ageing rapidly, with 1.2 million people currently aged 80 years or over; a figure projected to double within the next two decades (Australian Bureau of Statistics [ABS], 2023), significantly increasing the demand for aged care services, including nursing homes (Australian Institute of Health and Welfare [AIHW], 2024). In 2025 there were 196,313 people living in nursing homes across Australia, an increase of 6.7% from 5 years earlier (AIHW, 2024, 2025a).

The Royal Commission into Aged Care Quality and Safety highlighted the need for better access to allied health professions in nursing homes, including occupational therapy. Several recommendations were provided to improve the range, scope, and model of allied health service provision (Royal Commission into Aged Care Quality and Safety, 2021). Unfortunately, since 2022, and since the introduction of regulatory and funding mechanisms, there has been a reduction in both the median time residents in nursing homes receive allied health services and the associated expenditure (Gibson & Isbel, 2024), with negative consequences for allied health service provision and workforce capability (Allied Health Professions Australia, 2022).

Occupational therapy may be important to maintain function and support meaningful engagement in occupations, preserve dignity and enhance quality of life for people in nursing homes (Dancewicz & Bissett, 2020; Mitterfellner et al., 2024). There is moderate evidence to support the use of occupation‐based interventions and physical activities to improve occupational performance for adults in nursing homes, with further high‐quality intervention studies recommended, such as randomised controlled trials, to contribute to the evidence in this area (Mitterfellner et al., 2024). Residents have identified a need for services to prioritise urgent care needs, long‐term health habits, and social wellbeing (Meulenbroeks et al., 2024).

Quality of life is enhanced when activities align with individual interests and abilities (Russell, 2017), and occupational therapists are well placed to meet the diverse needs and priorities of people living in nursing homes (Calderone et al., 2022; World Federation of Occupational Therapists, 2019). However, current funding mechanisms limit access to occupational therapy, as well as the type and breadth of occupational therapy involvement.

Existing services focus on pain reduction, pressure care, and improving mobility (Rooney et al., 2024) with a notable lack of services aimed at psychosocial wellbeing and meaningful engagement. Furthermore, these services often appear fragmented and lack a holistic approach. Coordinated multidisciplinary teams are essential across all areas of healthcare, as they are well known to improve outcomes and prevent adverse events (Australian Commission on Safety and Quality in Health Care, 2021).

The Enhancing Allied Health for Older People (EAHOP) in Residential Care was a clinical trial (Isbel et al., 2025) using a pragmatic, non‐randomised, pre‐post methodology which delivered embedded, coordinated allied health disciplines to residents within a nursing home in Australia. The allied health professions included occupational therapy, physiotherapy, dietetics, speech pathology, pharmacy, optometry, and transdisciplinary allied health assistants, coordinated by a team leader who is an experienced registered nurse. The trial integrated with existing staff such as registered nurses, care coordinators, general practitioners, and allied health assistants, broader care home staff, and intermittent external providers.

The EAHOP trial enabled a responsive, needs‐based and resident directed occupational therapy service that focused on individually tailored interventions to improve occupational performance and satisfaction using a broader range of practice. The primary aim of this paper is to report on the implementation and outcomes of the occupational therapy service within the EAHOP trial.

## 2 METHODS

### 2.1 Study design

The occupational therapy component of the EAHOP trial followed a pragmatic, non‐randomised, pre‐post design, with an intervention period of up to 36 weeks per resident. A convergent mixed‐methods intervention evaluation was conducted (Fetters & Molina‐Azorin, 2020) with quantitative and qualitative data collected simultaneously and integrated during analysis (Fetters et al., 2013). Mixed methods were chosen to support triangulation of results, and to broaden understanding of the diverse factors that affect occupational therapy practice in nursing homes. Ethical approval was granted by the University of Canberra Human Research Ethics Commitee (Approval #11658).

### 2.2 Setting and participants

The study took place in a not‐for‐profit nursing home in Canberra which accommodates 100 residents and includes a dedicated memory support unit. All residents were eligible to participate in the EAHOP trial, as aligned with the inclusive and pragmatic positioning of the study. The EAHOP trial included a Stakeholder Committee that comprised two residents of the nursing home participating in the trial, the trial clinical liaison nurse, the site manager and the lead author. This group met monthly for the duration of the trial and advised the research team on all aspects of the intervention. Residents or their enduring power of attorneys or guardians consented to participate in the trial. Participants in the trial had their needs screened with the EAHOP clinical liaison nurse (team leader) and were recommended one or more of the six EAHOP allied health interventions. Participants then opted to undertake assessment from the recommended allied health disciplines and referrals were made to those disciplines, which included occupational therapy. The referral to occupational therapy was made following a discussion with the participant and/or their family member if the participant had any ADL, leisure or productivity challenges. The EAHOP team also liaised with the nursing home clinical and leisure staff to ask if the participant was experiencing any difficulties in these areas. Data reported in this paper relate only to the residents who received occupational therapy. Interviews were conducted with five participant groups to explore the processes and experiences of the trial: (1) occupational therapists on the EAHOP trial, (2) occupational therapy students, (3) nursing home support staff, (4) resident participant, and (5) family members. Written informed consent was obtained from all participants or their legal representatives.

### 2.3 Intervention

The occupational therapy interventions were informed by participants' goals relating to any meaningful occupations. An individualised intervention plan was developed, according to their goals and identified care needs. The model of care enabled transdisciplinary allied health assistants and occupational therapy students to deliver occupational therapy within their scope of practice. The duration of the intervention period varied depending on the needs and goals of the resident, ranging from 1 to 36 weeks. For example, one resident recovering from a total hip replacement post fall had a goal of putting on their own shoes and socks. This intervention period was brief to provide long handled aids and environmental modification to reduce the risk of further falls. Other residents with more complex needs were seen for 36 weeks.

### 2.4 Outcome measures

The Canadian Occupational Performance Measure (COPM) (Carswell et al., 2004) was used to assess participants' self‐rated satisfaction and performance in at least one occupational performance problem area identified by participants. Each problem was rated on a scale from 1 (*lowest*) to 10 (*highest*) for both performance and satisfaction, and scores were averaged to produce an overall score ranging from 1 to 10 for each component. Contemporaneous clinical records were collected and stored within a secure shared drive and included progress notes, formal reports, shared care plan documents, and scheduling spreadsheets. Following completion of the intervention period, a research assistant conducted a comprehensive chart audit of residents who received occupational therapy, which was cross‐checked for accuracy. Data were extracted and entered into a Microsoft Excel spreadsheet that recorded which interventions were provided to each resident.

As part of the overall EAHOP process evaluation, interviews were conducted as part of the trial evaluation to explore the trial processes, student placement experiences, and resident and family member experience. A secondary analysis of this data was conducted, with any text relevant to evaluating the occupational therapy component extracted. Interview guides for the process evaluation and student experience interviews are provided in Supporting Information S1. Resident and family member interviews were unstructured, guided by what participants deemed as important to their experience of living in a nursing home before and during the EAHOP trial. Experienced qualitative researchers (CP and AP) conducted the interviews following completion of the intervention period and were known to some interview participants. Interviews were recorded and transcribed using rev.com and checked for accuracy by AP. The interviewees were EAHOP clinicians (n = 4), occupational therapy students (n = 2), nursing home staff (n = 1), residents (n = 4), and family members (n = 2). Three complete interview transcripts from the occupational therapists and the occupational therapy students were included, which had a mean duration of 31 minutes (15–52 minutes). Transcripts of all interviews were reviewed and data relevant to the occupational therapy intervention were extracted.

### 2.5 Data analysis

Participant characteristics, implementation data, interventions delivered, and results from the COPM were analysed descriptively. Changes in COPM pre‐ and post‐measures were reported for performance and satisfaction, with a change of 2 points in both scores considered clinically meaningful (Carswell et al., 2004).

Qualitative data were analysed using manifest qualitative content analysis, following the five‐step methodology described by Graneheim and Lundman (2004) and conducted by authors AP and CP. This process involved (1) familiarisation with the interview transcripts, (2) identification of meaning units, (3) condensation of meaning units, (4) coding of condensed units, and (5) grouping of codes into subcategories and categories. The qualitative analysis was led by AP and CP. Two transcripts were independently coded to enhance interpretive depth, and findings were discussed collaboratively. Integration of findings involved comparing the quantitative results with the qualitative findings to provide a comprehensive understanding of the occupational therapy role and outcomes.

The trustworthiness of the qualitative findings was ensured by adhering to the criteria outlined by Graneheim and Lundman (2004). Credibility was achieved through prolonged engagement with the data, independent coding, and peer debriefing. Dependability was ensured by maintaining a clear audit trail of the analysis process. Transferability was supported by providing rich descriptions of the study context and participant characteristics. The trustworthiness of the qualitative data was further strengthened through triangulation with quantitative findings and reflective discussions among the research team.

## 3 RESULTS

### 3.1 Participant characteristics

Of the 36 residents who participated in the EAHOP trial, 32 (86%) opted to undergo an occupational therapy assessment and 27 (68%) were provided with interventions to address identified occupational performance issues. The remaining five residents, who participated in an assessment, were monitored by the occupational therapist throughout the 36‐week data collection period but did not require any occupational therapy. The average age of participants was 86 years, with a near‐equal gender distribution (52% female). Dementia and/or cognitive impairment was prevalent in 44% of the sample, with Psychogeriatric Assessment Scale interquartile ranges from 2.8 (minimal cognitive impairment) to 13.9 (moderate to severe cognitive impairment). See Table 1.

**TABLE 1:** Characteristics of the participating residents.

Characteristics	All (n = 36)	Occupational therapy (n = 27)
Age, mean (SD)	86 (6)	86 (6)
Female, n (%)	18 (50)	14 (52)
Cognition (PAS), median (IQR)	5.0 (1.8–10.5)	5.9 (2.8–13.9)
**Health conditions, n (%)**		
Anxiety/depression	15 (36)	19 (41)
Cardiac disease	31 (86)	11 (33)
Cerebrovascular disease/stroke	26 (72)	5 (19)
Parkinson's disease	6 (16.7)	5 (19)
Dementia	17 (47)	12 (44)
Hypertension	19 (52)	12 (44)
Incontinence	3 (8)	3 (11)

Two occupational therapists conducted assessments and delivered interventions during the trial. Both had extensive clinical experience (17 years and 31 years), in a wide range of clinical areas and health care settings, with special interests in neurological rehabilitation, dementia care, and aged care. The occupational therapists were employed and onsite for a total of 1.5 days per week for the 14‐month trial period. Two allied health assistants also delivered occupational therapy interventions within their scope of practice under the supervision of the occupational therapists. The allied health assistants were onsite for a total of 5 days per week to support the multidisciplinary team to deliver individual and group treatment sessions. Four final year occupational therapy students (three 4th year Bachelor of Occupational Therapy students and one 2nd year Master of Occupational Therapy student) at the University of Canberra completed an 8‐week block placement at separate times. Each student conducted assessments and delivered occupational therapy interventions within the aged care home.

### 3.2 Impact

The impact of the occupational therapy interventions is highlighted by the COPM results. See Table 2. Eighteen resident participants had an increase in occupational performance scores, of which 12 were clinically meaningful. Four participants maintained performance scores, and one decreased in performance. Twenty participants had an increase in occupational satisfaction scores, of which 12 were clinically meaningful. Three maintained satisfaction, and two decreased in satisfaction. Overall, participants demonstrated clinically meaningful improvements in both performance and satisfaction, with a median increase of 2 and 2.3 points, respectively.

**TABLE 2:** COPM results.

	Total (n = 25^a^)	
	Pre	Post
COPM		
Performance (median, IQR)	3.0 (2.2–4.2)	5.0 (3.8–6.0)
Satisfaction (median, IQR)	3.5 (2.4–5.0)	5.8 (3.6–6.5)

^a^ Twenty‐five participants completed pre‐ and post‐COPM measures. However, two participants had occupational problems identified and received occupational therapy to address them, declined to identify goals, and therefore did not have COPM results.

The impact of occupational therapy intervention on residents' lives was further reflected by qualitative data collected from students, residents and family. Residents were reported as being ‘more active, they're more engaging, and they are more emotionally stable’ (Student 2) in their daily lives. Moreover, a student described how when engaging in meaningful activities, they could see the ‘person lights back up again, and that was really lovely to see, I think there's a very stark difference’ (Student 1). This sentiment was echoed by residents, who reflected that re‐engagement ‘was a joy for us, but it was also worthwhile’ (Resident 4). Some residents reported the value of clinical support for their safety and independence. Family members also reported the value of occupational therapy for their relative ‘[OT] saw my mother as a whole person and took the time to get to know her and find out what was important to her. She also gave her hope that she could continue to improve’ (Family 1).

It was reported by the occupational therapists and students that the aged care home provided a complex and stimulating working environment for occupational therapists and the students reported being a valued part of a team with the EAHOP trial. They felt well supported by staff to build relationships with the residents, to understand the home's schedule and processes, and were comfortable to report back to the staff about residents' health. Overall, they described high satisfaction working in an aged care home through the EAHOP model.

Occupational therapy provided 29,535 minutes of care of which 17,310 minutes (59%) were direct care, with a median of 895 minutes of care per resident and a total of 612 occasions of service (OOS). Direct care was defined as ‘care that was provided face to face with the resident’. Indirect care included time spent doing reports, clinical notes, liaising with other health professionals and preparation for sessions.

The interprofessional allied health assistant provided 6480 minutes of care relating to occupational therapy, of which 5350 minutes (83%) was direct care with a median of 203 minutes per resident and a total of 199 occasions of service. Residents received a median of 3.9 minutes per day of occupational therapy interventions, including occupational therapy related interventions delivered by the allied health assistants. Participants received an average of 4.6 (SD 2.8) interventions and that was one or more of the interventions listed in Table 3.

### 3.3 Context

Residents and staff discussed some key gaps in usual practices within the nursing home that affected how the occupational therapy arm of EAHOP was conducted. This included gaps in usual practices that drove the need for occupational therapy, as well as impacting how the occupational therapist could operate within the home. The primary gap identified was a lack of personalised opportunities for meaningful engagement for residents, in particular, activities to keep themselves engaged during the day.

Well, uh you know, I apologise, but it's been boring a lot of the times, because you find, you look around to keep yourself busy…and after a while there's a limitation of what you can do to fill your time in. (Resident 1)

Whilst a leisure programme was run at the nursing home that provided opportunities for engagement, it was noted it was impossible to meet the needs of all residents in the home. As explained by one lifestyle officer, ‘there's a hundred residents, we can't do individualised activities for everyone, but we do try’ (Staff 1). Residents often experienced many barriers to independent engagement with meaningful activities, meaning that without staff facilitation, there are ‘a lot of people in residential aged care [who] were sitting there doing nothing or sitting there not doing the occupations that they wanted to do’ (OT 2).

The occupational therapists and students reflected on the need to adapt to the lives and schedules of residents, fluctuations in health, and the processes within the home such as personal and clinical care schedules, periods of contact restrictions, and weekly changes to the home activity schedule. As reported by a student, ‘I can't go in thinking I have eight hours in the day … it's really making the most of what times are going to be people times and what times are not going to be people times’ (Student 1).

Some students also reported the importance of not underestimating residents' capacity for therapy and activities. For example, one student reflected on a resident who they assumed would want to rest after returning home post cataract surgery. However, this resident requested to continue with their upper limb therapy session as planned.

It was reported that the nursing home provided a complex and stimulating working environment for occupational therapists, and the students reported being a valued part of a team with the EAHOP trial. They felt well supported by staff to build relationships with the residents, to understand the home's schedule and processes, and were comfortable to report back to the staff about residents' health.

Structural barriers to practice were noted to arise from the aged care system funding and safety regulations. Most prominent were the difficulties obtaining funding for equipment to support residents' identified needs, such as locating suitable bedside chairs, desk chairs, wheelchairs, or pressure cushions. The primary issue was limited availability of equipment onsite, as well as limited opportunities to source suitable equipment. A main factor in equipment procurement was restrictions on people living in the nursing home accessing the relevant government equipment scheme, closely followed by a high cost and limited availability of equipment for private hire or purchase. Typically, all recommended items required approval of staff or family members. The choice being removed from residents was described as an impediment to their autonomy, both in being unable to make financial decisions and to not receive equipment that would improve their safety, function and independence during activities of daily living.

Financial choices are up to the family, but it was more a question of why is it up to the family? I guess it really made me question why there aren't more supportive systems for people living in residential age facilities to be able to live in more comfort to be able to do more things, and that was a bit frustrating. (Student 1)

Moreover, many residents who had a Home Care Package in the community prior to entering the nursing home would have been able to access equipment through the package, but this access was removed once they moved into the home. Additionally, existing ‘one size fits all’ aged care regulations limited practice recommendations. An example was described where a resident with hemiparesis was unable to have bed rails necessary to enable independent bed mobility, as bedrails are considered both a restrictive practice and a safety hazard (Australian Commission on Safety and Quality in Health Care, 2021). A coinciding concern regarding equipment was observed through the unchecked disposal of equipment that was deemed faulty but easily repaired, or following the death of a resident, where the equipment could otherwise have been assigned to another resident.

### 3.4 Role of the occupational therapist

Various roles were fulfilled by the occupational therapists in the EAHOP trial, which fell under domains of clinical support, quality of life support, and nursing home service improvement. Within this role, five distinct categories began to emerge within the resident participant group that guided the intervention approach. These were dementia care (n = 7), degenerative neurological conditions (n = 6), palliative care (n = 1), reablement (n = 12), and slow‐stream rehabilitation (n = 1). Participants with neurological conditions (inclusive of slow‐stream rehabilitation) on average received the highest number of interventions. A detailed breakdown of the list and number of interventions given per category is contained in Supporting Information S2.

The clinical support domain consisted of occupational therapy assessments and interventions aimed at reablement, rehabilitation, maintenance of function, or prevention of unnecessary decline. This included exercise programmes, environmental modifications, and equipment prescription to support activities of daily living. Activity and cognitive engagement (n = 18, 67%) was the most provided intervention, followed by equipment selection (n = 15, 56%), family consultation (n = 15, 56%), and environmental modification (n = 14, 52%). See Table 3. Resident advocacy such as referrals to external service providers for specialist assessment and interventions was an additional role identified.

**TABLE 3:** Occupational therapy interventions delivered to participants.

Intervention summary	N (%)
Activity/cognitive engagement	18 (67)
Activity modification	7 (26)
Activities of daily living—personal	9 (33)
Activities of daily living—instrumental	4 (15)
Behaviours	2 (7)
Carer/care plan input	7 (26)
Community access	4 (15)
Environmental modification	14 (52)
Equipment	15 (56)
Falls prevention	6 (22)
Family consultation	15 (56)
Mobility/transfers	11 (41)
Pain management	2 (7)
Palliative comfort cares	1 (4)
Social engagement	6 (22)
Upper limb exercises/ranging	2 (7)

The physical environment of residents' rooms was described as not always being efficiently designed or set up to meet their needs. Although rooms were mostly modifiable, challenges described included making structural changes such as the need for bathroom sensor light installation, additional grabrails, raising sink heights, and difficulty overcoming regulatory and care requirements. For example, a bed placed beside the wall is a restrictive practice, and yet this change would allow for improved mobilisation and direct bathroom access for a person with Parkinson's disease.

Within the quality of life domain, residents described identifying opportunities to add or restore meaningful engagement into their lives. These activities were personalised based on residents' desires. Some examples of engagement activities included spending time outside of the nursing home, extended community walks, support to develop an autobiography, and outings such as to the botanical gardens, which used to be ‘one of the huge things in my life and my husband's life’ (Resident 4).

Occupational therapists described the importance of a person‐led process in their role, ensuring the interventions and support provided were in response to the residents' expressed need. The occupational therapists described education and training sessions with personal care workers regarding transfer techniques, equipment use, and monitoring implementation of interventions and recommendations. However, carer education was provided on an ad‐hoc basis, and a desire was expressed to include more formal and widespread training opportunities for nursing home staff.

## 4 DISCUSSION

The findings from this study demonstrate how occupational therapists can play a role in promoting the quality of life of people living in nursing homes through facilitating engagement in meaningful activities. Key findings include that occupational therapists can feasibly deliver a diverse range of interventions to support self‐care, mobility, leisure, and productivity occupations, which can produce clinically meaningful improvements in satisfaction and performance with occupations. People living in nursing homes have high care needs related to self‐care and reduced independence (AIHW, 2025b), which occupational therapists are well placed to address. This is typically where occupational therapy services have attempted to be directed, in addition to falls and pain management (Rooney et al., 2024). Currently, the primary areas of occupational therapy involvement do not routinely include interventions that directly enhance residents' psychosocial wellbeing, meaningful engagement and quality of life, with these areas often missed in nursing homes because of staffing challenges (Ludlow et al., 2020; Rooney et al., 2024).

The range of occupational therapy interventions in the EAHOP trial was greater than reported in the literature (Rooney et al., 2024). These included interventions such as Cognitive Stimulation Therapy (Woods et al., 2023), virtual cycling (D'Cunha et al., 2021), environmental modifications, workstation set up, electronic device accessibility, specialised wheelchair selection, community ambulation, gardening, voice recognition word processing, upper limb retraining, bed mobility and transfers, and staff and student education. The type and breadth of possible interventions demonstrated the wide range of occupational performance goals that can be meaningful to residents and the scope of occupational therapy possible in nursing homes when used to its full potential.

The embedded occupational therapy and multidisciplinary involvement within the EAHOP trial, provided more care than the reported national average of allied health in nursing homes. The Department of Health and Aged Care (2025) states that nationally, there is 3.9 minutes of allied health per resident, per day, inclusive of all involved allied health professions such as podiatry, dietetics, speech pathology, physiotherapy, and occupational therapy. There is no clear data on the amount, or funding source, of existing occupational therapy services in nursing homes in Australia.

Within the EAHOP trial, the median amount of occupational therapy delivered was 3.9 minutes, per resident per day; calculated over 7 days, in line with the Department of Health Disability and Ageing care minute reporting. Participants in this study received more occupational therapy than the median time reported nationally, as current figures are inclusive of all allied health service provision. A further consideration is that occupational therapy interventions were delivered over 5 days in the EAHOP study. Therefore, using 5 days as the denominator, the median time residents received occupational therapy was 5.1 minutes per day (Monday to Friday).

The impact of occupational therapy for residents was noted for maintaining personhood by supporting residents to re‐engage with activities that played important roles in their lives prior to entering a nursing home. There were clinically meaningful improvements in both performance and satisfaction within reported occupational performance problem areas, and generally positive feedback received within qualitative interviews. This builds upon and strengthens the evidence previously cited that occupational therapy may be beneficial in nursing homes with a further argument to expand the amount and scope of occupational therapy. This study has demonstrated that people living in nursing homes are not homogeneous, and their occupational needs will vary, highlighting the need for thoughtful, evidence‐informed, individualised occupational therapy.

The primary challenge experienced by the occupational therapists related to equipment, raising concerns about equity and autonomy for residents. This was seen for residents who were identified as requiring equipment; however, access to this was impacted by significant costs or the inability to purchase the equipment by the nursing home or family members. Autonomy loss once entering a nursing home is a key concern for many older adults (Kalaitzidis & Harrington, 2018; Moilanen et al., 2021), and the inability to access equipment is an important case study emphasising the impact of this issue. Equity is at the forefront of occupational therapy practice (Egan & Restall, 2022), and the ability to access necessary equipment for independence, safety, and meaningful engagement in daily lives once entering a nursing home is important.

### 4.1 Limitations and future directions

This study had a small sample size and was based in one nursing home in Australia, limiting its generalisability to other settings. The occupational therapists delivering occupational therapy services in the trial were experienced clinicians, which may have influenced the outcomes. Future research should aim to replicate the model of care used in the trial in other settings, with a larger sample in geographically diverse areas and should consider addressing the challenges around equipment funding and access for residents with high needs. An initial consideration could be to create equitable and sustainable equipment processes for maintenance and storage of equipment that is no longer in use by residents. This would require staff with expertise regarding equipment, which further supports the need for embedded occupational therapy services.

The EAHOP model of care involved occupational therapy students and allied health assistants to deliver occupational therapy within their scope and under the supervision of an occupational therapist. Given the acute shortage of allied health in nursing homes, this type of service delivery should be further investigated. Future work should include ensuring funding for occupational therapy and allied health more broadly to provide the types of interventions people living in nursing homes require.

## 5 CONCLUSION

This study has shown that the EAHOP occupational therapy service implemented a broader range of practice, embedded into a multidisciplinary model of care within a nursing home. Residents in this trial reported clinically meaningful improvements in occupational performance and satisfaction and valued the opportunity to maintain a level of independence and re‐engage with meaningful occupation. Occupational therapy is currently underfunded and therefore underutilised within nursing homes. This study provides a blueprint to support the implementation of embedded occupational therapists, as well as transdisciplinary allied health assistants and occupational therapy students within nursing homes.

## AUTHOR CONTRIBUTIONS

*Discipline‐specific input to the intervention*: Brie Bobinskas, Claire Pearce, and Stephen Isbel. *Study design and methodological*: Stephen Isbel, Helen Holloway, Diane Gibson, Kasia Bail, and Nathan D'Cunha. *Critical manuscript review*: Stephen Isbel, Brie Bobinskas, Diane Gibson, Helen Holloway, Claire Pearce, Nathan D'Cunha, Kasia Bail, and Alice Pashley. *Write up and manuscript preparation*: Alice Pashley, Brie Bobinskas, and Stephen Isbel.

## CONFLICT OF INTEREST STATEMENT

Author Stephen Isbel is an Associate Editor for the *Australian Occupational Therapy Journal*. Author Claire Pearce is an Editorial Board member for the *Australian Occupational Therapy Journal*.

## ETHICS STATEMENT

This study was registered on ACTRN12624000028505 12 January 2024 with the Australian and New Zealand Clinical Trials Registry.

## DECLARATION OF USE OF ARTIFICIAL INTELLIGENCE

The plain language summary was entered into Co‐Pilot (TM) with the prompt to rewrite to a Flesch Kincaid reading level no greater than 8. The output was checked, refined, and confirmed by the authors before inclusion.

## POSITIONALITY STATEMENT

The authors of this paper include a registered, experienced occupational therapy clinician (BB), two registered occupational therapy clinical academics (SI and CP), a registered clinical nurse academic (HH) with extensive experience in nursing homes, three academics with extensive aged care experience in nursing (KB), dementia care (NDC) and policy (DG), and a post‐doctoral clinical academic (AP). All authors completed their clinical training and/or higher degrees in Australia. All authors have a specific interest in caring for people in nursing homes and we acknowledge that this has influenced the aims of the research and how the research was carried out.

## TERMINOLOGY

The term ‘nursing home’ used in this paper refers to a residential setting where nursing care is given to residents alongside other allied health and care requirements. The terminology used internationally varies in different countries. The Australian government currently uses the terms ‘residential aged care’, or ‘aged care home’ (Australian Government, 2026), whereas other countries use ‘care homes’ and ‘nursing homes’ (Carehome UK, 2026), or ‘skilled nursing facilities’, ‘long term care’, or ‘residential facilities’. The World Health Organisation (WHO) uses ‘long‐term care at a residential facility’ (WHO, 2025), where the OECD uses ‘long‐term care’ (OECD, 2026). We have chosen the term ‘nursing home’ as it is in common use and is readily understood in a variety of geographic contexts.

## Supporting information


**Data S1.** Supporting Information.

**Data S2.** Supporting Information.

## Data Availability

The data that support the findings of this study are available on request from the corresponding author. The data are not publicly available due to privacy or ethical restrictions.
