# Supplementary material for: Implementation and outcomes of a novel occupational therapy service in a nursing home
Source: Aust Occup Ther J. 2026 Apr 27;73(3):e70089. doi: 10.1111/1440-1630.70089 (PMC13112328; doi:10.1111/1440-1630.70089)
Supplement: Supplementary file 1 — Data S1. Supporting Information. [file AOT-73-0-s002.docx]

# **Supplementary file 1**

Process evaluation interview guide

Can you tell me about your role at Calvary/in relation to EAHOP? Were you previously working with Calvary prior to EAHOP?

*For EAHOP staff:*

Can you give me an overview of how you think [AH Discipline] has been implemented at Calvary?

What elements of the [AH discipline] approach do you think worked best? Can you provide an example of this in relation to a particular resident?

Was there any elements that did not add value? Can you provide an example?

What factors influenced how the [AH discipline] approach was implemented?

Can you give me an overview of how you think the allied health program overall has been implemented at Calvary?

What elements of the allied health program do you think worked best? Can you provide an example of this in relation to a particular resident?

Was there any elements that did not add value? Can you provide an example?

What factors influenced how the allied health program was implemented?

What about the Allied Health program do you think is essential to continue in the future?

Would you change anything about the program?

Student placement experience interview guide

Thank you again for agreeing to be interviewed. As you know we have been running an allied health program for residents here at Calvary Haydon including people with dementia or cognitive impairment. This is the first time something like this has been done so we are keen to get the thoughts and opinions of students who have undertaken a placement at Calvary, about the program.

First of all, could you please let me know your role and how long your placement was?

- What do you think you learnt about working in aged care and specifically, with people with dementia?
- Can you share your reflections on the value of your profession’s role within aged care
- What do you think and feel about working in aged care and/ or with older adults in the future?
- Can you share an experience from placements that stood out for you that had an impact on you either personally or professionally
