# Supplementary material for: Implementation and outcomes of a novel occupational therapy service in a nursing home
Source: Aust Occup Ther J. 2026 Apr 27;73(3):e70089. doi: 10.1111/1440-1630.70089 (PMC13112328; doi:10.1111/1440-1630.70089)
Supplement: Supplementary file 2 — Data S2. Supporting Information. [file AOT-73-0-s001.docx]

## Supplementary file 2. Occupational Therapy interventions

| **Area** | **Occupational Performance Problems** | **Interventions** |
| --- | --- | --- |
| Dementia care | Falls  Wandering  Evening aggression  Situational distress  Social isolation | Cognitive Stimulation Therapy (1:1 and group)  Purposeful activity engagement e.g. help set table, fold clothes, gardening  Virtual cycling  Environmental modification (room orientation)  Community walking program  Carer support for evening schedule  Referral and advocacy to link with suitable volunteer |
| End-stage degenerative neurological conditions  e.g.  Parkinsons Disease    Multiple Sclerosis | Frequent falls  Poor engagement in quiet leisure  Limited mobility  Worsening posture  Pressure care issues  Unable to go outside with family  Isolation | Environmental modification for falls prevention  Bathroom adaptation (rails, obstacle removal, task adaptation)  Specialised equipment prescription  e.g. tilt in space wheelchair for postural alignment, pressure care, and improved socialisation (not looking down at floor)  Bedroom pressure, bed positioning, and transfer equipment  Adapted physical activity program  Virtual cycling  Advocacy /referral to external services  GP and Medical Specialists  TAD ACT: for accessible desk, chair  ACT Equipment Service (specialised wheelchair) |
| Palliative care | Pressure care and positioning issues  Worsening pain (immobility) Social isolation (if family not present)  Reduced garden access with family or friends | Pressure care and equipment prescription  Soft splint (finger contractures)  Cognitive-sensory gentle ROM (upper limb and lower limb) for comfort cares  Care plan recommendations / carer support |
| Reablement for frail aged and low vision | High falls risk  Limited mobility around room  Isolation with prolonged time in room  Reduced activity engagement  Reduced ability to manage daily activities | Environmental modification for falls prevention and improved function   - Room reorganisation and improved access - Rail installation; contrasting colour rails - Re-position toilet roll holder - Extra lighting: bedroom, bathroom, dining - Instigate call bell repair   Equipment assessment and prescription   - Transfer equipment - Adjust OTF, provide insert - Install pull out clothes drying rack - Adapted desk set up - Visual aids   Pressure ulcer ax, equipment prescription, nursing / carer support  Individual exercise and activity engagement program  Seating assessment |
| Slow-stream rehabilitation | Recent hospital discharge due to medical condition  No pathway to access hospital outpatient or centre-based community rehabilitation services (referral)  Altered level of function  Reduced independence with bed mobility, transfers, self-care tasks, and mobility in room and around facility  Unable to access public hospital outpatient or community rehabilitation services | Individual exercise program:   - postural control and dynamic sitting balance - upper limb strengthening - self-care functional retraining - functional mobility (in room, around facility) - wheelchair assessment and prescription - Spasticity management: strengthening, stretching, splinting and serial casting; referral for Botulinum Toxin injections   Environmental adaptation (wheelchair access)  Equipment assessment and prescription   - Identify and facilitate repair of faulty equipment (pressure mattress, walker, wheelchair brakes and footplate) - Wheelchair adaptations e.g. extended handle for brake   Preventative interventions   - footplate wedge to block ankle inversion (contracture would impact transfers) - pressure and bedside chair assessment   Equipment delivery set up and liaison with hospital OT (joint session with equipment company  Education and monitoring  e.g. Post total hip replacement:  functional transfers, environmental adaptations, equipment prescription (sock donner) |
|  |  |  |
